# Supplementary material for: More than 2,500 coding genes in the human reference gene set still have unsettled status
Source: bioRxiv. 2024 Dec 9:2024.12.05.626965. Preprint. [Version 1] doi: 10.1101/2024.12.05.626965 (PMC11661123; doi:10.1101/2024.12.05.626965)
Supplement: Supplement 1 [file media-1.pdf]

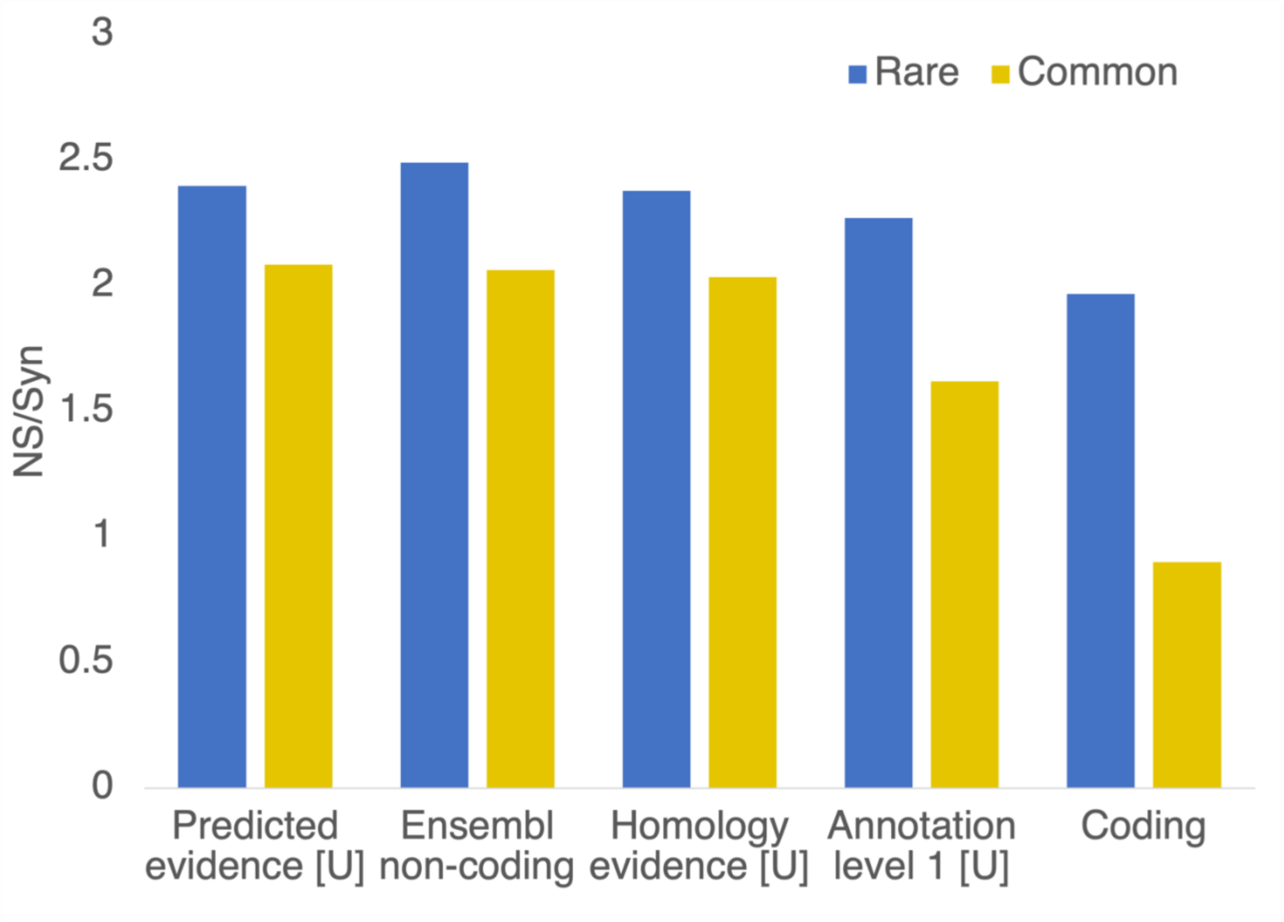

Supplementary Figure 1
The NS/Syn ratios calculated for potential non-coding features excluded from the analysis. Features marked with a “U” are annotator evidence codes that come directly from the UniProtKB database. Ensembl non-coding genes are those marked as non-coding, non-functiona or pseudogene. Coding gene NS/Syn ratios (from genes under purifying selection) are included as a control.

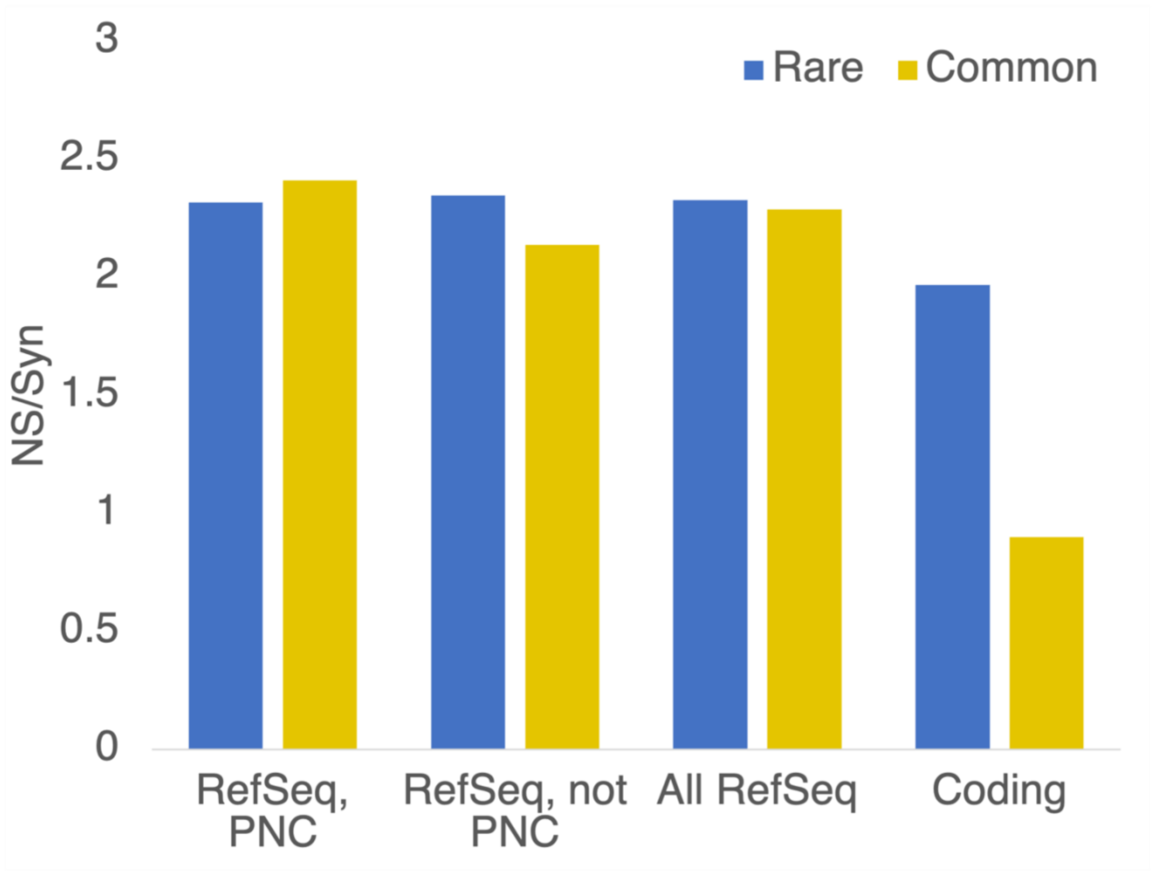

Supplementary Figure 2
The NS/Syn ratios calculated for predicted RefSeq genes not in the intersection. “RefSeq, PNC” are those RefSeq coding genes not in the intersection of the three sets that are tagged with one of the three applicable potential non-coding features. “RefSeq, not PNC” are RefSeq coding genes not in the intersection that are not tagged with potential non-coding features. “All RefSeq” are RefSeq coding genes not in the intersection. Coding gene NS/Syn ratios (from genes under purifying selection) are included as a control.

```
HIG2DB HUMAN      MA-----------TLGFVTPEAPFESSKPPIFEGLSPTVY-S-NPEGFKEKFLRKTRENP
MARMOSET          MA-----------TPGPVTPEASLEPSKPPVIEGFSPTLY-S-KPESFKEKFIRKVRENP
MACAQUE           MA-----------TPGPVIPEVPFEPSKPPVIEGLSPTVY-R-DPETFKEKFLRKTRENP
HIG2DA HUMAN      MA-----------TPGPVIPEVPFEPSKPPVIEGLSPTVY-R-NPESFKEKFVRKTRENP
RAT               MA-----------APGPVSPEAPFDPSKAPVIEGFSPTVY-T-NPEGFKEKFIRKTRENP
MOUSE             MA-----------APGPVSPEAPFDPSKPPVIEGFSPTVY-S-NPEGFKEKFIRKTRENP
HAMSTER           MA-----------APRPVSSEAPFDPSQTPVIEGFTPTVY-S-NPESFKDKFIRKTRENP
BAT               MT-----------TPGPVTPGTPFEPSQPPVVEGISPSVY-S-TTESFKEKFLRKTRENP
GUINEA PIG        MA-----------TPGPVTPEAPFKPSQPPVIEGFNPSVH-I-HQEGFKEKFLRKTRENP
PIG               MA-----------TPGPATPEAPFEPSHPPVIEGFSPTVY-S-TSESFKKKFIRKTRENP
TARSIER           MA-----------TPGPVTPEVPFEPSQPPVIEGFSPSVY-G-NPESFKEKFLRKTRENP
CAT               MA-----------VPGPVTPEAPFEPSQPPVIEGFSPSVY-S-TPESFKEKFLRKTRENP
DOG               MA-----------APGPVTPGAPFEPSQPPVIEGFSPSVY-S-PPESFKEKFLRKTRENP
FOX               MA-----------APGPVTPGAPFEPSQPPVIEGFSPSIY-S-TPESFKEKFLRKTRENP
POLAR BEAR        MA-----------APGPVTPGAPFEPSQPPVIEGFSPSIY-N-TQESFKEKFLRKTRENP
SEAL              MA-----------APGPVTPGAPFEPSQPPVIEGFSPSIY-S-TQESFKEKFLRKTRENP
DOLPHIN           MA-----------TPGPVTPEAPFEPSQPPVIEGFSPSVY-S-TSESFKEKFLRKTRENP
SHEEP             ME-----------TPGRVTPEAPFEPSQPPVIEGFSPSVY-S-TSEGFKEKFIRKTRENP
COW               ME-----------TPGRVTPEAPFEPSQPPVIEGFSPSVY-S-TSESFKEKFIRKTRENP
KILLFISH          MAATTRPSEPESHVKEQPPGALPFVLSQPPVIEGFRQSPK-V-KDETFKEKFIRKTKENP
SNAILFISH         MA-GATAVSELTPAAKSPAAFAPFDFSQPPDIEGFSRLPA-A-RDETFKEKFLRKTKENP
TROPICAL FROG     --------------------------MAHPEIEGFTPSST-Y-GDEGFKSKFIRKVKENP
```

```
AFRICAN FROG        --------------------------MAQPEIEGFTPS-T-Y-TSEGFKGKFIRKVKENP
SHARK               ---------------MAAPVREEVSLSKPPVIEGFHPAPR-P-REESFRDKFIRKTSENP
COD                 MAAASTPAVVNSPI-TQQSGGMPFDFSKPPVIEGFTPVSR-R-KDETFKEKLLRKTKENP
TROUT               MAAATTPVVPEQSASTT--HLPMLDISKPPVIDGFTPLSR-P-REETFQEKFMRKSKENP
CAVE FISH           MAVASSAVQEERAGRTALPGAAAFNPGSPPVIEGFTPLPR-Q-KEESFKEKFLRKTKENP
ZEBRAFISH           MATAAAPVSPDQPGKSA-SPPVLLDLSQPPVIEGFSPTSR-T-REEGFKDKFIRKTKENP
CATFISH             MAAASSRVEQASPVKTAPPIAAGFDLSDPPVIDGFTPLSR-P-REEGFKDKFIRKTKENP
HERRING             MAASPTVVSQDQAANAASQGPIPFDISKPPIIEGFTPLPR-H-REEGFKDKFIRKTKENP
MILKFISH            MAAASTPVERDQVVKTPQSGPVPFDISKPPVIEGFTPLPR-A-KEEGFKDKFIRKTKENP
CHICKEN             ---------------MAAGPPPPLEPIPLPVY-----------RDEGFADKFRRKTRENP
ZEBRA FINCH         ---------------MAAGPPPPLEPSPLPTF-----------PEEGFTEKFVRKTRENP
BARN SWALLOW        ---------------MAAGPPPPLDPIPLPTF-----------TEEGFAEKFLRKTRENP
WALL LIZARD         ---------------MAQSAPPPFDPNNPPLIEGFTPTAY-H-PEEGFGDKFRRKTRENP
ANOLE LIZARD        ---------------MTQAPPPPFDPSRPPLIEGFQPGAF-QRREEGFADKFLRKTRENP
                                                 *               * *  *: **  ***

HIG2DB HUMAN        VVPIGFLCTAAVLTNGLYCFHQGNSQCSRLMMHTQIAAQGFTIAAILLGLAATAMKSPP-
MARMOSET            MVPIGCLATATALGYGLYCFHKGHSRRSQLMMRTRIAAQGFTIAAILVGLGVTSMKSRP-
MACAQUE             VVPIGCLATVAALTYGVYSFYRGDSRRSQLMMRTRIAAQGFTVTALLLGLAVTAMKSRP-
HIG2DA HUMAN        VVPIGCLATAAALTYGLYSFHRGNSQRSQLMMRTRIAAQGFTVAAILLGLAVTAMKSRP-
RAT                 MVPIGCLGTAAALTYGLYCFHRGQSHRSQLMMRTRIAAQGFTVVAILLGLAASTMKSRS-
MOUSE               MVPIGCLGTAAALTYGLYCFHRGQSHRSQLMMRTRIAAQGFTVVAILLGLAASAMKSQA-
HAMSTER             MVPIGCLGTAAALSYGLYCFHRGQSHRSQIMMRTRIAAQGFTVVAILLGLAASAMKSRS-
BAT                 MVPLGCLSTAAALTYGLYCFHRGQSQRSQLMMRTRIAAQGFTIVAILVGLAASALKSRP-
GUINEA PIG          MVPIGCLGTAAALTYGLYCFHQGHSQRSQFMMRTRIAAQGFTVAAILLGLAASAMKSRS-
PIG                 MVPIGCLGTASALTYGLYCFHRGQSQRSQLMMRTRIAAQGFTIVVILVGLAASTMRSRP-
TARSIER             MVPIGCLGTAAALTYGLYCFHRGHSQRSQLMMRTRIAAQGFTVAAILLGLAASALKSRP-
CAT                 MVPIGCLGTAAALTYGLYCFHRGQSHRSQLMMRTRIAAQGFTVAAILLGLAASAMRSRS-
DOG                 MVPVGCLGTAAALTYGLYCFHRGQSHRSQLMMRTRIAAQGFTVAAILLGLAASAMKSRS-
FOX                 MVPIGCLGTAAALTYGLYCFHRGQSHRSQLMMRTRIAAQGFTVAAILLGLAASAMKSRS-
POLAR BEAR          MVPIGCLGTAAALTYGLYCFHRGQSHRSQLMMRTRIAAQGFTVAAILLGLAASAMKSRS-
SEAL                MVPIGCLGTAAALTYGLYCFHRGQSHRSQLMMRTRIAAQGFTVAAVLLGLAASAMKSRS-
DOLPHIN             MVPIGCLGTAAALTYGLYCFHRGQSQRSQLMMRTRIAAQGFTVVAILMGLAASTMKSRP-
SHEEP               LVPIGCLGTAAALTYGLYCFHRGQSQRSQLMMRTRIAAQGFTIVAILVGLAASTLKSRP-
COW                 LVPIGCLGTAAALTYGLYCFHRGQSQRSQLMMRTRIAAQGFTIVAILVGLAASTLKSRP-
KILLFISH            FVPIGCLGTTGMLMYGLRSFHQGKTKQSQMFMRGRIFAQGFTVVAIIVGIFATALKPKQ-
SNAILFISH           FVPIGCLGTAGALVYGLRAFNQGKTRQSQLMMRGRIFAQGFTVVAIIAGVFITAMKPKQ-
TROPICAL FROG       FVPIGCLATAGALTYGLISFKQGKTQQSQLLMRTRILAQGFTVAAIMFGVVMTAMKPRIT
AFRICAN FROG        FVPIGCLATAGALTYGLISFKQGKTRQSQLLMRTRILAQGFTVAAIMVGVVMTALKPSET
SHARK               FVPLGMLGTAGALTYGLIAFNHGKTRHSQLSMRARIFAQGFTIVAIVVGVVATTLKPK-
COD                 FVPIGCLGTAGALAYGLRAFHQGKTRQSQMLMRGRIFAQGFTVFAIIFGVVATQLKPKQ-
TROUT               FVPIGCLGTAGALMYGLRAFKQGKTRQSQLLMRGRIFAQGFTVVAIIFGVFTTALKKD--
CAVE FISH           FVPIGCLGTAGALTYGLRAFKHGKTHQSQMLMRTRIFAQGFTVIAIIVGVAATALKSKQ-
ZEBRAFISH           FVPIGCLGTAGALIYGLGAFKQGKTRQSQLLMRTRIFAQGFTVVAIIVGVAATALKAKP-
CATFISH             FVPIGCLGTAGALIYGLRAFKQGKTRQSQLLMRTRIFAQGFTVVAIIVGVAAAALKPRQ-
HERRING             FVPIGCLGTAGALIYGLRAFKMGKTRQSQLSMRMRIFAQGFTVVAIIVGVASTALKPKQ-
MILKFISH            FVPIGCLGTAGALIYGLSAFRQGKTRQSQLLMRARIFAQGFTVVAIIVGVATTALKSK-
CHICKEN             LVPLGCLCTLGVLTYGLISFKRGNTRHSQLMMRARVVAQGFTVAALLGGMVATALRARS-
ZEBRA FINCH         LVPLGCLCTVSVLVYGIICFKRGQTRRSQLMMRARVIAQGCTFAALLGGMVATALKSRQ-
BARN SWALLOW        MVPLGCLCTVGVLAYGVICFKKGNTRRSQLMMRARVVAQGFTIASVVGGMMATAIRSRQ-
WALL LIZARD         LVPIGCLGTAGVLAYGLICFKKGNTLQSQRMMRARVLAQGFTVAAILVGVVVASMKPKK-
ANOLE LIZARD        LVPVGCLGTAGVLTYGLICFKRGNTHQSQIMMRARILAQGFTVAALVVGVVVTALKPKK-
                    .**:* * *   * **: .*  *.   **  ** *: *** *.  :: *:     :
```

Supplementary Figure 3. Alignment of human *HIGD2B* with vertebrate *HIGD2A* homologues
Alignment of human *HIG2DB* with 35 vertebrate *HIGD2A* paralogues. *HIGD2A* sequence is shown in bold with the 29 residues that are completely conserved across the 35 *HIGD2A* in blue. *HIGD2B* sequence is shown in bold with residues that are different from completely conserved *HIGD2A* residues in red, residues that are radically different from highly conserved *HIGD2A* residues in orange and other residues that are radically different in green.
